# Exploratory evaluation of pharmacodynamics, pharmacokinetics and safety of rivaroxaban in children and adolescents: an EINSTEIN-Jr phase I study

**DOI:** 10.1186/s12959-018-0186-0

**Published:** 2018-12-04

**Authors:** Dagmar Kubitza, Stefan Willmann, Michael Becka, Kirstin Thelen, Guy Young, Leonardo R. Brandão, Paul Monagle, Christoph Male, Anthony Chan, Gili Kennet, Ida Martinelli, Paola Saracco, Anthonie W. A. Lensing

**Affiliations:** 1 Bayer AG, Global Drug Discovery – Clinical Sciences, Clinical Pharmacology Cardiovascular, Aprather Weg 18a, Gebäude 429, 42113 Wuppertal, Germany; 2 Research and Clinical Sciences, Bayer AG, Wuppertal, Germany; 3 Children’s Hospital Los Angeles, University of Southern California Keck School of Medicine, Los Angeles, CA USA; 4 Department of Paediatrics, Division of Haematology/Oncology, The Hospital for Sick Children, University of Toronto, Toronto, Canada; 5 Department of Haematology Royal Children’s Hospital, Department of Paediatrics, University of Melbourne, Murdoch Children’s Research Institute, Melbourne, Australia; 6 Thrombosis & Haemostasis Unit, Department of Paediatrics, Medical University of Vienna, Vienna, Austria; 7 McMaster Children’s Hospital/Hamilton Health Sciences Foundation Pediatric Thrombosis and Hemostasis, Hamilton, Canada; 8 National Hemophilia Center & Thrombosis Institute, Sheba Medical Center, Ramat Gan, Israel; 9 A.Bianchi Bonomi Hemophilia and Thrombosis Center, Fondazione IRCCS Ca’ Granda - Ospedale Maggiore Policlinico, Milan, Italy; 10 Pediatric Hematology, University Hospital Città della Salute e della Scienza, Torino, Italy; 11 Clinical Development, Bayer AG, Pharmaceuticals, Wuppertal, Germany

**Keywords:** Developmental hemostasis, Pharmacodynamics, Pharmacokinetics, Rivaroxaban, Venous thromboembolism

## Abstract

**Background:**

The EINSTEIN-Jr program will evaluate rivaroxaban for the treatment of venous thromboembolism (VTE) in children, targeting exposures similar to the 20 mg once-daily dose for adults.

**Methods:**

This was a multinational, single-dose, open-label, phase I study to describe the pharmacodynamics (PD), pharmacokinetics (PK) and safety of a single bodyweight-adjusted rivaroxaban dose in children aged 0.5–18 years. Children who had completed treatment for a venous thromboembolic event were enrolled into four age groups (0.5–2 years, 2–6 years, 6–12 years and 12–18 years) receiving rivaroxaban doses equivalent to 10 mg or 20 mg (either as a tablet or oral suspension). Blood samples for PK and PD analyses were collected within specified time windows.

**Results:**

Fifty-nine children were evaluated. In all age groups, PD parameters (prothrombin time, activated partial thromboplastin time and anti-Factor Xa activity) showed a linear relationship versus rivaroxaban plasma concentrations and were in line with previously acquired adult data, as well as in vitro spiking experiments*.* The rivaroxaban pediatric physiologically based pharmacokinetic model, used to predict the doses for the individual body weight groups, was confirmed. No episodes of bleeding were reported, and treatment-emergent adverse events occurred in four children and all resolved during the study.

**Conclusions:**

Bodyweight-adjusted, single-dose rivaroxaban had predictable PK/PD profiles in children across all age groups from 0.5 to 18 years. The PD assessments based on prothrombin time and activated partial thromboplastin time demonstrated that the anticoagulant effect of rivaroxaban was not affected by developmental hemostasis in children.

**Trial registration:**

ClinicalTrials.gov number, NCT01145859.

## Introduction

In adults, rivaroxaban is associated with similar efficacy and a significantly lower rate of major bleeding compared with low molecular weight heparin (LMWH) followed by dose-adjusted vitamin K antagonist [VKA] therapy) in patients with acute venous thromboembolism (VTE) [1].

Although adult data are the basis for planning pediatric trials, it is important to assess potential differences between children and adults in terms of both pharmacokinetics (PK) and pharmacodynamics (PD), because these may affect pediatric dosing algorithms. The distinct differences in the coagulation system between adults and children, referred to as developmental haemostasis [2], may affect treatment with anticoagulants in children. Specifically, the concentrations of coagulation factors are different in children and change as they grow [2].

Therefore, it is important to understand whether rivaroxaban treatment results in different PD responses in children, compared with adults. To address this question, previous in vitro spiking experiments were performed in plasma obtained from children and neonates, with the results suggesting that PD responses to rivaroxaban across a range of age groups were similar to those in adults [3, 4]. In these in vitro spiking experiments, the prothrombin time (PT) and activated partial thromboplastin time (aPTT), which have been frequently used in adults to describe the PD properties of rivaroxaban and their relationship to plasma concentrations, were assessed to establish whether they displayed similar properties in children [3, 4].

Physiological differences in children also affect the absorption, distribution, metabolism and excretion properties of drugs [5]. Our physiologically based pharmacokinetic (PBPK) model, which was used to predict pediatric rivaroxaban doses, takes into account growth and variability in anthropometrics (e.g. body height, weight and body mass index), anatomy (e.g. organ weight) and physiology (e.g. blood flow rates) for the respective ages, as well as the maturation of metabolism and excretion processes [6].

The purpose of the rivaroxaban EINSTEIN-Jr program is to establish bodyweight-specific dosing regimens, aiming at exposures similar to the rivaroxaban 20 mg once-daily dose used for the treatment of VTE in adults. A comprehensive development program – consisting of several phase I, II and III studies – is underway to assess the safety, efficacy and PK/PD of rivaroxaban in children. The program was approved by both the Pediatric Committee of the European Medicines Agency and the United States Food and Drug Administration.

The main objective of this phase I study was to collect PK/PD data in children aged 0.5–18 years who received a single bodyweight-adjusted rivaroxaban dose.

## Methods

### Subjects and study design

**Fig. 1:**
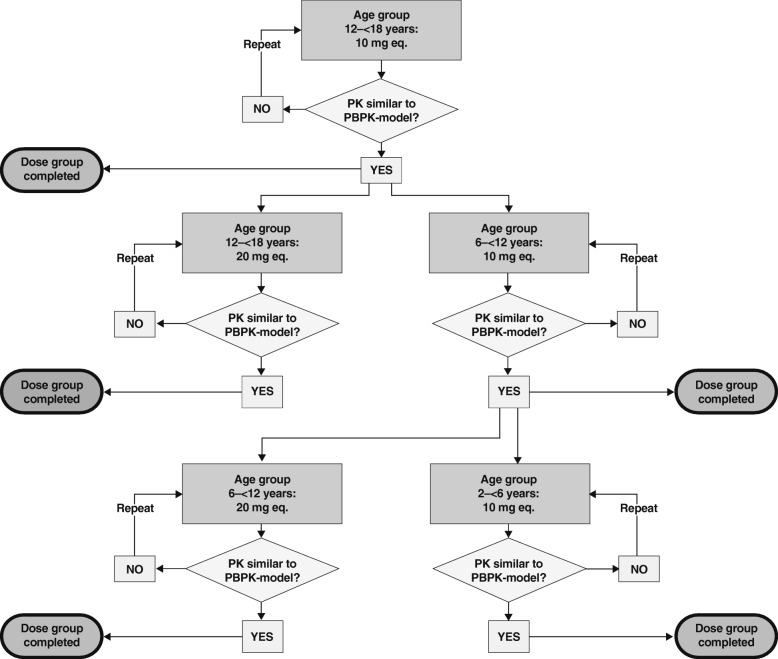
Staggered approach; decision tree of the Data Monitoring Committee. PBPK, physiologically based pharmacokinetic; PK, pharmacokinetics

This was a multinational, multicentre, single-dose, phase I study. Children were eligible if they had completed standard anticoagulant treatment (i.e., unfractionated heparin, low molecular weight heparin, fondaparinux or vitamin K antagonists) for acute VTE at the time of rivaroxaban administration. The main inclusion and exclusion criteria are listed in Table 1. Children were stratified by age: 0.5–2, 2–6, 6–12 and 12–18 years. Children received a single bodyweight-adjusted oral dose of rivaroxaban (either as a tablet or oral suspension) that was derived from PBPK model predictions that targeted equivalent exposures to rivaroxaban doses of 10 mg or 20 mg in adults (10 mg-equivalent or 20 mg-equivalent). Dose linearity was tested between the rivaroxaban 10 mg-equivalent dose and 20 mg-equivalent dose (i.e. the licensed dose for treatment of VTE in adults after initial treatment with rivaroxaban 15 mg twice-daily for 21 days) [7]. The study was conducted in a staggered fashion, starting with the oldest children and the lower dose first. An independent Data Monitoring Committee evaluated the safety and PK/PD data from all children before allowing the study to proceed to the next age or dose/formulation cohort (Fig. 1).

**Table 1:** Main inclusion and exclusion criteria

Inclusion criteria	Exclusion criteria
• Children aged 0.5–< 18 years at the time of administration of the study drug, who had completed treatment for VTE and were considered to be at risk of recurrence, but with no current need for anticoagulation • Children who could take oral medication • Written informed consent (and age-appropriate assent) as per local requirements • Clinically stable children who could be treated on an ambulatory basis • A normal prothrombin time and activated partial thromboplastin time	• Any condition requiring ongoing anticoagulation • Known bleeding disorder • Any major or clinically relevant bleeding event during previous VTE treatment • Abnormal coagulation test results within 10 days prior to study drug administration • Medical disorder, condition or history of such that would impair the child’s ability to participate or complete this study in the opinion of the investigator or sponsor

*VTE* venous thromboembolism

The study aimed to achieve exposures within the adult range; however, it was accepted that exposures in individual children may be below the adult reference range. Low exposure was not a concern because all children had completed VTE treatment at study entry. Since off-label use of preliminary established pediatric rivaroxaban dose regimens could put children at risk for thrombotic and bleeding complications, we refrain from reporting the applied rivaroxaban dose regimens.

The protocol was approved by the Institutional Review Board at each participating centre. The parent or legal guardian provided written, informed consent and the subject signed the assent form, if applicable.

### Rivaroxaban administration

A single body-weight-adjusted dose of rivaroxaban was administered up to 2 h after intake of a meal as either a tablet or an oral suspension, depending on age. Children aged ≥12 years received rivaroxaban as a tablet, those aged 6–12 years received rivaroxaban as either a tablet or an oral suspension at the discretion of the investigator, and children aged < 6 years received rivaroxaban as an oral suspension. The oral suspension of rivaroxaban was either administered as an undiluted suspension (directly into the mouth) or as a diluted suspension through mixing with a defined volume of non-sparkling liquid at a target dilution ratio of 1:2 to 1:10 (volume of rivaroxaban suspension:volume of dilution liquid).

### Collection of blood samples

Blood samples for PD/PK analyses were collected within specified time windows for each age group. Central laboratory coagulation tests (PT, aPTT and anti-Factor Xa activity) were performed before study drug administration. Central laboratory PK blood sampling followed by PD blood sample tests were carried out at different time points after study drug administration depending on the age group: at 90 min–5 h and 20–24 h in children aged 0.5–2 years and 2–6 years, with an additional measurement at 8–12 h in those aged 2–6 years. In children aged 6–12 years and 12–18 years, measurements were taken at 30–90 min, and 2–5, 8–12 and 20–24 h, with an additional measurement at 4–8 h in those aged 12–18 years. Samples were taken by venepuncture, central venous line or peripheral catheter. If blood was withdrawn from a catheter, a sufficient amount of blood had to be discarded prior to taking the PD samples to avoid contamination of samples from heparinised lines. Blood samples for PK were stored at or below − 15 °C and analysed at the DMPK Bioanalytical Laboratory of Bayer (Wuppertal, Germany) within 4 weeks of sampling. Blood samples for PD analysis were frozen at − 20 °C, and PT and aPTT were analysed within 4 weeks of sampling at the Pathology and Clinical Pathology Laboratory at Bayer (Wuppertal, Germany). Blood samples for anti-Factor Xa activity were stored at − 15 °C and analysed within 4 weeks of arrival at the Laboratory for Translational Assay Technology at Bayer (Wuppertal, Germany).

### Pharmacodynamic assays

PT, aPTT and anti-Factor Xa activity were measured on an ongoing basis at a central laboratory. The PD data were analysed for their relationship to their respective rivaroxaban plasma concentration in the various age groups and compared with adult data. The adult reference population were healthy volunteers aged 19–40 years (*n* = 171) receiving a single dose of rivaroxaban 10 mg, or aged 19–40 years (*n* = 101) receiving a single dose of rivaroxaban 20 mg, who had been enrolled in the adult phase I rivaroxaban program.

#### Prothrombin time assay

The PT assay was performed in accordance with the manufacturer’s instructions using an STA Compact coagulation analyser (Diagnostica Stago S.A.S., Asnières sur Seine Cedex, France) with the use of STA NEOPLASTIN CI PLUS 10® reagent (Diagnostica Stago S.A.S.) and an International Sensitivity Index (ISI) of around 1.3.

#### Activated partial thromboplastin time assay

The aPTT assay was performed using a STA Compact coagulation analyser with the use of STA C.K. Prest 5® reagent (Diagnostica Stago S.A.S.). The test was performed using standard methods in accordance with the manufacturer’s instructions.

#### Anti-factor Xa assay

Anti-Factor Xa activity was determined using a photometric method in accordance with the recommendations of the manufacturer (Technoclone GmbH, Vienna, Austria) and with specific calibrators and controls (Technoclone GmbH) for calibration. The limit of rivaroxaban detection was 0.1 ng ml^− 1^, with a lower limit of quantification of 14.5 ng ml^− 1^ and an upper limit of quantification of 433.3 ng ml^− 1^. The intra-day coefficients of variation were 1.92, 1.48 and 9.77% for the low, medium and high concentrations, respectively; the inter-day coefficients of variation were 4.44, 3.12 and 1.72% for the low, medium and high concentrations, respectively.

### Pharmacokinetic parameters

Rivaroxaban plasma concentrations were determined using high-performance liquid chromatography–tandem mass spectrometry after solid/liquid extraction [8]. The calibration range of the procedure was from 0.500 μg l^− 1^ (lower limit of quantification) to 500 μg l^− 1^ (upper limit of quantification). Mean inter-assay accuracy of back-calculated concentrations (except lower limit of quantification) in the calibrators ranged between 93.3 and 104.5% and precision was ≤4.4%. Rivaroxaban plasma concentrations were calculated from the chromatographic raw data. PK parameters (such as area under the plasma concentration–time curve zero to 24 h [AUC], maximum plasma concentration [C_max_] and minimum plasma concentration measured 20 to 24 h after rivaroxaban administration [C__24h_]) were determined as described in the accompanying study [9].

### Safety and tolerability

Each child was followed up for at least 7 days after rivaroxaban intake for the occurrence of adverse events (AEs); for another 23 days thereafter, the investigator had to report serious AEs.

### Statistical analysis

For any preliminary inspection of dose-dependent exposure behaviour, the observed plasma concentration–time data were compared with PBPK predictions for the respective age groups or to data in adults [9].

Statistical evaluation was performed using the software package SAS release 9.2 (SAS Institute Inc., Cary, NC, USA). All variables were analysed by descriptive statistical methods. The number of data available, and missing data, as well as mean, standard deviation, minimum, median and maximum were calculated for metric data. Frequency tables were generated for categorical data.

Summary statistics for subjects who received rivaroxaban are presented separately according to dose, formulation and age group. Individual listings of AEs are provided. The incidences of treatment-emergent AEs (TEAEs) and drug-related AEs are summarised by dose, formulation and age group, using Medical Dictionary for Regulatory Activities version 18 terms.

Baseline was defined as the last observation prior to administration of the study drug. Baseline for centrally assessed anti-Factor Xa activity was not measured and was assumed to be zero. E_trough_ for the PD characteristics was defined as the minimum value in the time interval 20–24 h for all subjects and values. E_max_ for the PD characteristics was defined as the maximum value in the time intervals excluding 20–24 h for all subjects.

Relative changes from baseline were considered for PT and aPTT, and absolute changes from baseline were considered for anti-Factor Xa activity.

Pool reference data were based on BAY 59–7939 phase I trials up to 11 November 2011 (unpublished data on file, Bayer AG, Leverkusen, Germany). Selected subjects were of white race, were valid for PK analyses and had received a single dose of either rivaroxaban 10 mg or 20 mg under fasted or fed conditions and did not use any other medication. Subjects were between 18 and 55 years old, had a body mass index > 18 or < 30 kg/m2 and had serum creatinine, alanine aminotransferase, and total bilirubin values lower than the upper limit of normal.

Owing to the sparse sampling scheme and the limited number of subjects who can be enrolled in a paediatric study (for ethical and feasibility reasons), a visual comparison of the spread of individual PT, aPTT, anti-Factor Xa activity and plasma concentration data in comparison with adult data was considered most meaningful for the assessment of potential differences between children and adults.

## Results

From November 2010 to June 2015, a total of 59 children from 18 sites in 7 countries (Australia, Austria, Canada, France, Israel, Italy, United States) were enrolled and received rivaroxaban. All subjects were valid for the PK, PD and safety analyses. Demographic and baseline characteristics by age group and dose equivalence are listed in Table 2.

**Table 2:** Demographics and baseline data

		12–18 years		6–12 years				2–6 years		0.5–2 years		Total (*N* = 59)
		Tablet 10 mg eq. (*n* = 4)	Tablet 20 mg eq. (*n* = 5)	Tablet 10 mg eq. (*n* = 4)	Suspension 10 mg eq. (*n* = 11)	Tablet 20 mg eq. (*n* = 4)	Suspension 20 mg eq. (*n* = 5)	Suspension 10 mg eq. (*n* = 11)	Suspension 20 mg eq. (*n* = 5)	Suspension 10 mg eq. (*n* = 6)	Suspension 20 mg eq. (*n* = 4)	
Sex	Male	3 (75)	1 (20)	3 (75)	5 (45)	2 (50)	5 (100)	6 (55)	2 (40)	3 (50)	3 (75)	33 (56)
	Female	1 (25)	4 (80)	1 (25)	6 (55)	2 (50)		5 (45)	3 (60)	3 (50)	1 (25)	26 (44)
Race	White	4 (100)	4 (80)	2 (50)	7 (64)	3 (75)	5 (100)	7 (64)	3 (60)	5 (83)	4 (100)	44 (75)
	Black	–	–	–	–	–	–	–	1 (20)	–	–	1 (2)
	Asian	–		–	1 (9)	–	–	1 (9)	1 (20)	–	–	3 (5)
	Hispanic	–	1 (20)	2 (50)	1 (9)	1 (25)	–	2 (18)	–	–	–	7 (12)
	Missing	–	–	–	2 (18)	–	–	1 (9)	–	1 (17)	–	4 (7)
Age (years)	Mean ± SD	16.0 ± 1.4	14.8 ± 1.9	10.0 ± 0.8	7.5 ± 1.5	8.5 ± 1.9	9.2 ± 1.8	3.4 ± 1.2	3.6 ± 1.5	0.5 ± 0.5	0.5 ± 0.6	6.8 ± 4.9
	Median	16.5	15.0	10.0	7.0	9.0	9.0	3.0	4.0	0.5	0.5	6.0
	Range	14.0–17.0	12.0–17.0	9.0–11.0	6.0–11.0	6.0–10.0	7–11.0	2.0–5.0	2.0–5.0	0.5–1.0	0.5–1.0	0.5–17.0
Weight (kg)	Mean ± SD	68.6 ± 8.0	56.9 ± 8.9	40.2 ± 6.7	29.8 ± 4.9	37.3 ± 12.2	35.0 ± 7.9	17.8 ± 6.5	16.0 ± 3.3	8.9 ± 1.7	9.6 ± 2.2	29.5 ± 18.3
	Median	67.5	58.0	41.6	28.2	34.9	32.0	15.3	16.0	9.4	9.8	27.7
	Range	61.5–77.8	42.2–64.4	31.5–46.1	24.0–38.2	27.0–52.5	28.5–47.8	11.0–29.1	11.0–20.0	6.2–11.0	6.7–11.9	6.2–77.8
Height (cm)	Mean ± SD	173.7 ± 9.0	165.9 ± 11.9	141.6 ± 6.0	125.3 ± 8.7	133.1 ± 14.8	139.5 ± 9.8	92.5 ± 21.6	99.1 ± 11.3	72.0 ± 7.6	75.7 ± 6.3	117.7 ± 34.0
	Median	170.0	167.4	141.7	125.0	132.6	136.0	98.0	103.0	73.5	75.8	118.0
	Range	167.6–187.0	149.5–180.1	136.0–147.0	114.3–143.5	116.0–151.0	132.0–156.7	35.0–118.0	83.5–111.0	59.0–80.0	68.0–83.0	35.0–187.0
BMI (kg/m^2^)	Mean ± SD	22.8 ± 2.6	20.6 ± 1.7	20.1 ± 3.4	19.0 ± 2.1	20.8 ± 4.8	17.8 ± 2.1	16.9 ± 3.2	16.1 ± 1.1	17.1 ± 1.0	16.5 ± 1.4	18.5 ± 3.0
	Median	21.8	19.9	19.6	18.4	19.2	16.9	15.8	15.8	17.2	16.9	17.8
	Range	20.8–26.6	18.9–22.7	16.8–24.2	15.9–23.0	17.0–27.9	15.4–20.6	14.6–25.7	15.1–18.0	15.6–18.3	14.5–17.4	14.5–27.9

Data are n (%) unless stated otherwise

*BMI* body mass index; *eq.*, equivalent, *SD* standard deviation

### Pharmacodynamics

#### Prothrombin time and rivaroxaban plasma concentrations

**Fig. 2:**
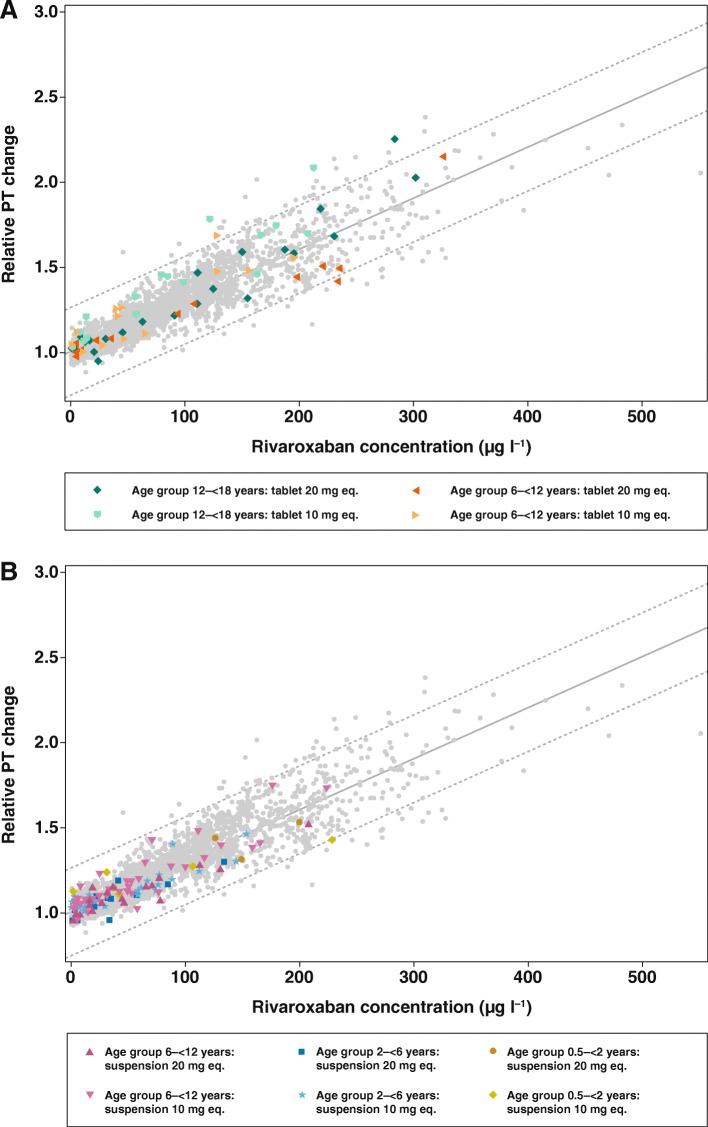
Prothrombin time changes from baseline for all ages and all doses with (**a)** rivaroxaban tablet and (**b**) rivaroxaban suspension. Light grey dots are data points from healthy adult volunteers; coloured dots represent individual data from children. A linear relationship was assumed for the concentration–response curve. 99% prediction intervals were used to depict the variability in the pooled data. The two reference populations consisted of healthy adult volunteers from phase I trials, who received either a single dose of rivaroxaban 10 mg (age 18–40 years, *n* = 171), or a single dose of rivaroxaban 20 mg (age 18–40 years, *n* = 101). PT change from baseline (x-fold) is individual PT at a distinct time point, divided by individual PT prior to drug administration. eq., equivalent; PT, prothrombin time

In all age groups, PT changes from baseline showed a linear relationship versus rivaroxaban plasma concentrations (Fig. 2), and the data were similar to those of healthy adult volunteers receiving a single dose of rivaroxaban 10 mg or 20 mg. All individual PT data points were within the prediction limits or within the individual distribution of adult data points regardless of dose. There was no apparent difference between the tablet and oral suspension formulations in the correlation between PT and rivaroxaban plasma concentrations in the age group 6–12 years.

#### Activated partial thromboplastin time and rivaroxaban plasma concentrations

**Fig. 3:**
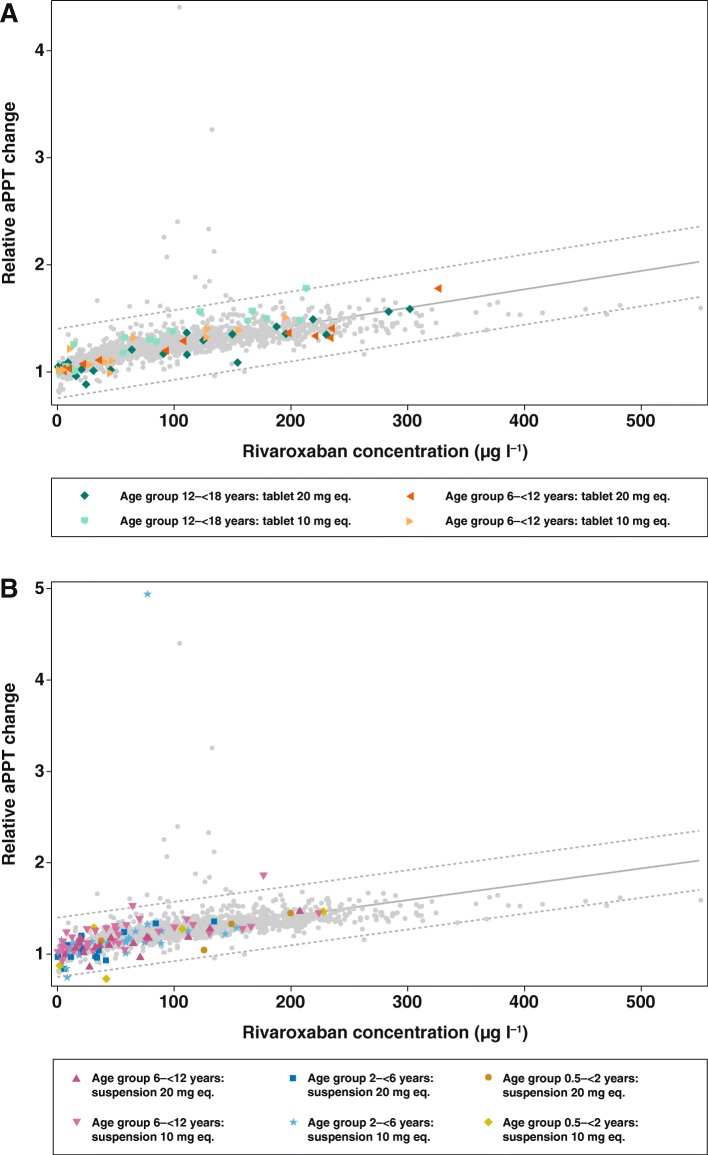
Activated partial thromboplastin time changes from baseline for all ages and all doses with (**a**) rivaroxaban tablet and (**b**) rivaroxaban suspension. Light grey dots are data points from healthy adult volunteers; coloured dots represent individual data from children. A linear relationship was assumed for the concentration–response curve. 99% prediction intervals were used to depict the variability in the pooled data. The two reference populations consist of healthy adult volunteers from phase I trials, who received either a single dose of rivaroxaban 10 mg (age 18–40 years, *n* = 171), or a single dose of rivaroxaban 20 mg (age 18–40 years, *n* = 101). aPTT change from baseline (x-fold) is individual aPTT at a distinct time point, divided by individual aPTT prior to drug administration. aPPT, activated partial thromboplastin time; eq., equivalent

In all age groups, aPTT changes from baseline showed a linear relationship versus rivaroxaban plasma concentrations (Fig. 3), and data were similar to those obtained from healthy adults. All individual aPTT data points were within the prediction limits or within the individual distribution of adult data points, regardless of dose. There was no apparent difference between the tablet and oral suspension formulations in the correlation between aPTT and rivaroxaban plasma concentrations in the age group 6–12 years. The correlation graphs for aPTT were less steep than for PT, reflecting the lower sensitivity of the aPTT assay to rivaroxaban compared with the PT assay.

#### Anti-factor Xa activity and rivaroxaban plasma concentrations

**Fig. 4:**
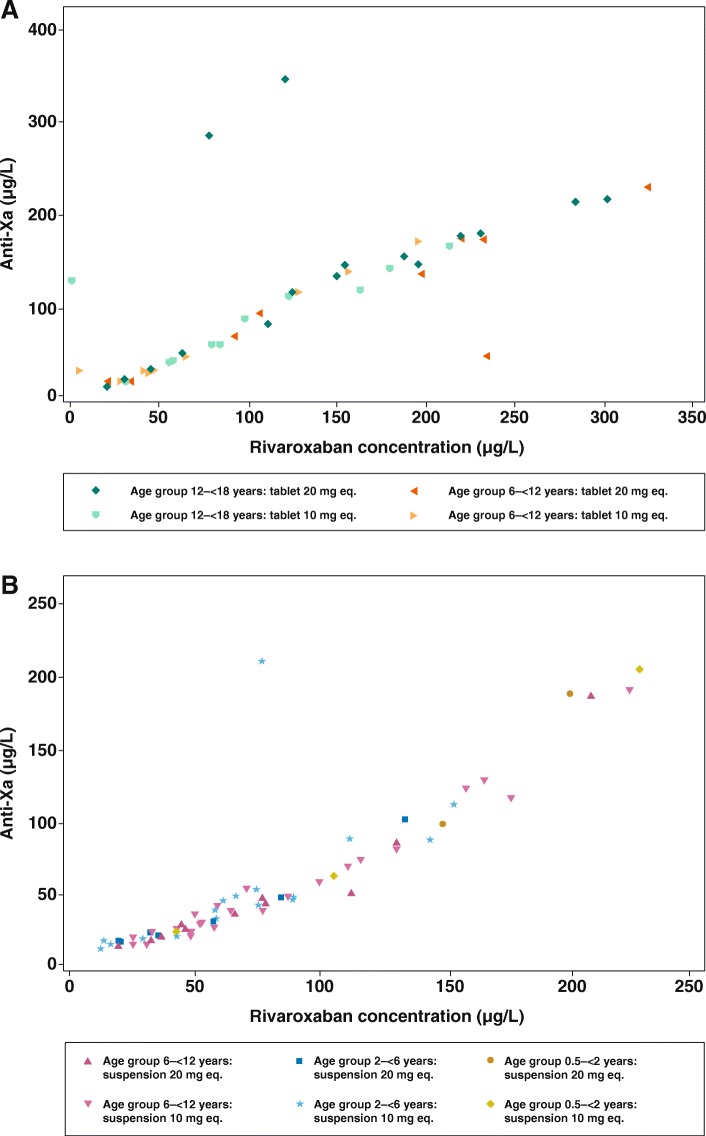
Anti-Factor Xa activity versus plasma concentrations; rivaroxaban 10 mg and 20 mg-equivalent doses for all age groups (*n* = 59) with (**a**) rivaroxaban tablet and (**b**) rivaroxaban suspension. Anti-Factor Xa activity within the 24 h after administration of rivaroxaban; eq., equivalent

The data seem to indicate a linear relationship of anti-Factor Xa activity and plasma concentrations for all age groups (Fig. 4). However, data are limited and must be supplemented by further investigations in future trials. In addition, it has to be noted that some individual data points deviated from this linearity.

### Pharmacokinetics

**Fig. 5:**
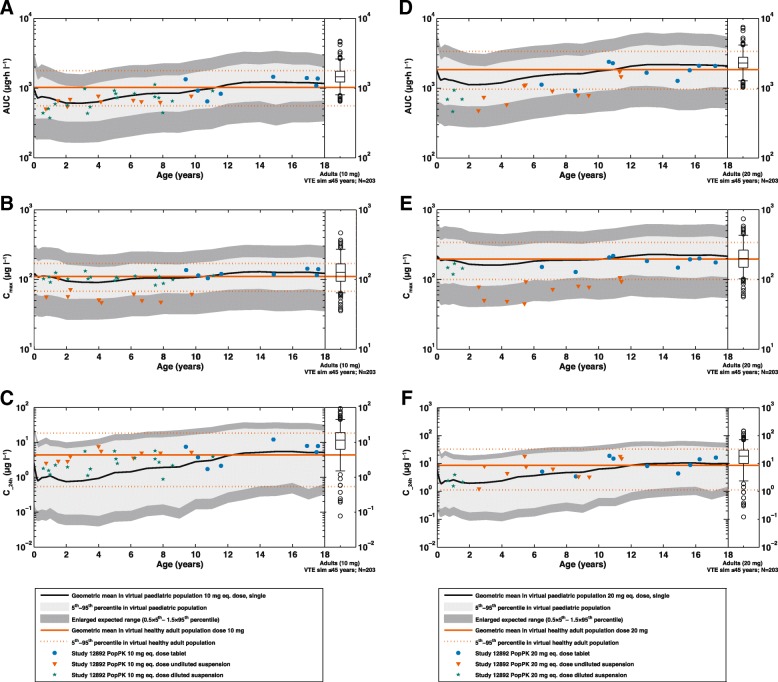
**a** Area under the plasma concentration–time curve from time 0–24 h (AUC); (**b**) maximum plasma concentration (C_max_) and (**c**) minimum plasma concentration measured 20–24 h after rivaroxaban administration (C__24h_) versus age for rivaroxaban 10 mg dose equivalent; and (**d**) AUC, (**e**) C_max_ and (**f**) C__24h_ versus age for rivaroxaban 20 mg dose equivalent, for children and adolescents between 6 months and 18 years of age, compared with the corresponding PBPK model predictions for children and adolescents, simulated via PBPK modelling and population PK modelling (box-whisker plot indicating the percentiles 5, 25, 50, 75 and 95). The grey shaded region shows the interval between the 5th and 95th percentiles of the population simulations with the PBPK model, the dashed shaded region shows an enlarged expected range due to uncertainties of some physiological parameters in children that may affect the absorption and clearance. The upper limit of this expanded range was calculated as 1.5 x 95th percentile and the lower limit was calculated as 0.5 x 5th percentile of the PBPK model estimate. PBPK, physiologically based pharmacokinetic; PK, pharmacokinetics

The PK of rivaroxaban in children were as expected. Irrespective of dose or formulation, the parameter values for AUC, C_max_ and C__24h_ obtained via population PK modelling for the entire age range (0.5–< 18 years) were completely contained within the enlarged expected ranges based on PBPK modelling (Fig. 5). The results demonstrated that the rivaroxaban pediatric PBPK model, used to predict the doses for the individual body weight groups, was confirmed by parameter values for AUC, C_max_ and C__24h_ obtained in this study.

The observed values for AUC, C_max_ and C__24h_ were also in good agreement with corresponding results obtained using the healthy adult PBPK model and the adult VTE treatment population PK model. However, although the starting dose aimed for an exposure that was within the adult exposure range, an observed exposure below the adult reference range was accepted in individual children to ensure that the adult exposure ranges after rivaroxaban 10 mg and 20 mg were not exceeded. As a result, children younger than 6 years had a tendency towards lower exposures than observed in adult VTE treatment patients, as was predicted based on paediatric PBPK modelling. For the rivaroxaban 20 mg-equivalent dose, a trend towards lower than average predicted AUC and C_max_ values in children aged < 12 years was additionally seen, suggesting absorption limitation for the higher dose equivalent in younger children. Further details are provided in the related model validation paper [9].

### Safety and tolerability

Rivaroxaban was well tolerated across all age groups. There were no deaths, major bleeding events or non-major clinically relevant bleeding events observed. Overall, 16 of the 59 children (27.1%) reported at least one TEAE. Most TEAEs were of mild (10/59; 16.9%) or moderate (5/59; 8.5%) intensity. Of these, one child, a 17-year-old boy with several prothrombotic risk factors had a pelvic venous thrombosis 7 days after the single-dose of rivaroxaban. There were no TEAEs or serious AEs caused by procedures required in the protocol. No differences in the frequency of AEs across dose, formulation or age groups were apparent. TEAEs considered to be related to rivaroxaban were reported in 4 of the 59 children (7%): abdominal discomfort (mild intensity, no treatment required), dyspepsia (mild intensity, no treatment required), allergic dermatitis (moderate intensity, remedial therapy given) and urticaria (mild intensity, remedial therapy given). All of these drug-related TEAEs resolved. Laboratory parameters (i.e., hemoglobin, blood platelets, creatinine, total and direct bilirubin and alanine aminotransferase) and vital signs (i.e., blood pressure and heart rate) were not affected by rivaroxaban administration.

## Discussion

A single body-weight-adjusted dose of rivaroxaban, as predicted by PBPK modelling, targeting equivalent exposures to the fixed dose administration of rivaroxaban 10 mg and 20 mg used in adults [9], was used to compare PK/PD profiles of children aged 0.5–18 years to adults.

The PK data confirmed the predictions of the PBPK model that target an exposure of single-dose rivaroxaban in children similar to that in healthy adults receiving single doses of rivaroxaban 10 mg or 20 mg. Comparing AUC, C_max_ and C__24h_ parameters in children with those in an adult VTE population showed that the data from children < 10 years old were in the lower range of the adult data. This was an expected finding because the doses were targeting the adult reference range; however, because of uncertainties of the prediction, lower doses were chosen in this trial. This uncertainty in the predictions was specifically applicable to children < 10 years old. Because only children who had completed their treatment for VTE were eligible for this study, lower exposure compared with adults was not a therapeutic issue. Based on this concept, it was clear that the doses used in this phase I study will need to be adapted for future phase II studies.

The PD effects of rivaroxaban in children were also in agreement with the adult data. The correlation between PT changes from baseline and plasma concentrations was linear and within the reference range of healthy adult volunteers in both dose groups. Importantly, this was a consistent finding over the complete age range, from 0.5–18 years, and for both tablets and oral suspension. Similar results were observed for aPTT. The correlation between anti-Factor Xa activity and plasma concentration was linear, as described by the manufacturer of the assay. It should be noted that the anti-Factor Xa assay is displayed in μg/L as plasma concentration, but it is nevertheless a coagulation assay. As such, the anti-Xa assay can be influenced by pre-processing procedures (e.g. blood draw technique, too long time until measurement), which may contribute to falsely high or low values. In contrast, the determination of plasma concentration by HPLC (which is the gold standard) is much more stable and less prone to pre-processing procedures. The results of this study were in line with the in vitro data, which showed that rivaroxaban had no age-related effect on PT, aPTT or anti-Factor Xa activity. A dose-dependent prolongation of PT and aPTT was found in response to rivaroxaban across the complete age range tested in vitro [3, 4]. Together, the results of the in vitro and phase I studies in children demonstrated that the anticoagulant effect of rivaroxaban is not affected by developmental hemostasis in the age range of 0.5 to 18 years. In addition, these data demonstrated that bodyweight-adjusted dosing of rivaroxaban in children achieves predictable PK/PD profiles irrespective of the dose or formulation. Consequently, the PK–PD relationship for rivaroxaban in children is similar to that in adults.

Although anticoagulants have been studied in both adults and children, paediatric studies have included much smaller numbers of subjects [10]. The aim of pediatric studies in general is to achieve PD effects similar to those in adults, because this is considered to preserve safety and efficacy, and it is generally considered not feasible to perform large randomised trials in children.

Several studies have evaluated the pharmacology of unfractionated heparin, LMWH and warfarin in pediatric populations [10]. These studies indicated that higher bodyweight-based dosing was necessary for the youngest patients in order to achieve similar PD effects to those observed in adults, likely as a result of an inverse correlation between body-weight-normalised clearance of these drugs [10]. However, these anticoagulants have several shortcomings in children. For example, the anti-Factor Xa activity of LMWHs is dependent on its binding to antithrombin. However, antithrombin levels can vary widely in children according to their age, as well as with underlying medical conditions [11, 12]. As a result, the usually predictable dosing profile of LMWHs seen in adults may be affected and the anticoagulant effect reduced. Another key practical limitation of LMWHs is the need for parenteral administration, which may be challenging for both children and their parents and may negatively impact on compliance and acceptance of treatment duration [13]. VKAs are only available as tablets and, therefore, there is no age-appropriate formulation for younger children, who are not able to swallow tablets. Crushing tablets and dividing them to facilitate administration may result in inaccurate dosing. In addition, frequent blood sampling is necessary for monitoring.

PK, PD and safety data relating to the use of newer anticoagulants in children are also limited. Data from three small phase II clinical trials of dabigatran in children aged < 12 months (*n =* 8), 1–12 years (*n =* 18) and 12-18 years (*n* = 9) are available [14–16]. Children aged < 12 years received a single dose of dabigatran oral liquid formation based on an age- and body weight-adjusted nomogram yielding an exposure equivalent to 150 mg in adults; adolescents received dabigatran capsules, administered twice-daily for 3 days, at a weight-adjusted dose targeting 80–100% of the 150 mg dose used in adults. Across all three studies, dabigatran was generally well tolerated and the PK/PD profile was similar to the established profile in older patients with VTE [14–16]. The FondaKIDS trial showed that fondaparinux (0.1 mg kg^− 1^ once-daily) in children resulted in a similar PK profile to that seen in adults receiving standard dosing. However, a relatively small number of subjects were enrolled (*n* = 24) with few subjects in each age cohort, thereby limiting the extrapolation of results to the broader pediatric population [17]. Two intravenous, direct thrombin inhibitors, bivalirudin and argatroban, have also been evaluated in pediatric populations. In a study of 16 infants (aged < 6 months) and another study of 18 children (aged 0.5–18 years) with VTE, bivalirudin was shown to have an acceptable safety profile and predictable PD effects [18, 19]. A further study of 110 children undergoing cardiac catheterisation showed that the PK/PD response of bivalirudin in prophylactic doses was similar in children and in adults [20]. Argatroban was evaluated in 18 children requiring non-heparin anticoagulation and was found to provide adequate levels of anticoagulation and to be well tolerated [21].

Our study had some limitations. First, this was a relatively small study because it is very difficult to enrol children in clinical studies, particularly phase I studies where there is no direct therapeutic benefit for the individual child. However, because the aim of the trial was to determine the PK and PD of rivaroxaban in children, this small number of subjects was acceptable. Second, the trial covered a large age range, which meant that several factors may contribute to the variability for both PK and PD parameters. Third, as with most pediatric trials, sparse sampling schemes had to be used. Guidelines specify pediatric blood sample volume limits ranging from 1 to 5% of total blood volume over 24 h and up to 10% of total blood volume over 8 weeks [22]. These limitations underline the necessity of comprehensive investigations and of understanding all the relevant factors that may influence the PK/PD properties of anticoagulants in children.

## Conclusions

Bodyweight-adjusted, single-dose rivaroxaban has predictable PK/PD profiles in children across all age groups. The PK data are in good agreement with the PBPK model predictions and support the use of the model for further dose and dosing regimen optimisation. The PD assessments are in agreement with the results of a previous in vitro study and demonstrat that the anticoagulant effect of rivaroxaban is not affected by developmental hemostasis in the age range of 0.5–18 years. Our data support the EINSTEIN-Jr phase II and III studies, which will assess the safety and efficacy, as well as the PK and PD properties, of rivaroxaban for the treatment of VTE in the pediatric population.

## Abbreviations

aPTT: Activated partial thromboplastin time
AUC0–24: Area under the plasma concentration–time curve from time 0–24 h
C_24h: Plasma concentration 24 h after rivaroxaban administration
Cmax: Maximal plasma concentration
Emax: Maximum observed pharmacodynamics measurement
PBPK model: Physiologically based pharmacokinetic model
PD: Pharmacodynamic
PK: Pharmacokinetics
PT: Prothrombin time
tmax: Maximum plasma concentration
VTE: Venous thromboembolism

## References

[1] Prins MH, Lensing AWA, Bauersachs R, van Bellen B, Bounameaux H, Brighton TA (2013). Oral rivaroxaban versus standard therapy for the treatment of symptomatic venous thromboembolism: a pooled analysis of the EINSTEIN-DVT and PE randomized studies. Thromb J.

[2] Monagle P, Ignjatovic V, Savoia H (2010). Hemostasis in neonates and children: pitfalls and dilemmas. Blood Rev.

[3] Attard C, Monagle P, Kubitza D, Ignjatovic V (2012). The *in vitro* anticoagulant effect of rivaroxaban in children. Thromb Res.

[4] Attard C, Monagle P, Kubitza D, Ignjatovic V (2014). The *in-vitro* anticoagulant effect of rivaroxaban in neonates. Blood Coagul Fibrinolysis.

[5] Kearns GL, Abdel-Rahman SM, Alander SW, Blowey DL, Leeder JS, Kauffman RE (2003). Developmental pharmacology-drug disposition, action, and therapy in infants and children. N Engl J Med.

[6] Willmann S, Becker C, Burghaus R, Coboeken K, Edginton A, Lippert J (2014). Development of a paediatric population-based model of the pharmacokinetics of rivaroxaban. Clin Pharmacokinet.

[7] Bayer AG (2018). Xarelto® (rivaroxaban) Summary of Product Characteristics.

[8] Rohde G (2008). Determination of rivaroxaban – a novel, oral, direct factor Xa inhibitor – in human plasma by high-performance liquid chromatography-tandem mass spectrometry. J Chromatogr B Analyt Technol Biomed Life Sci.

[9] Willmann S, Thelen K, Kubitza D, Lensing AWA, Frede M, Coboeken K, et al. Pharmacokinetics of rivaroxaban in children using physiologically based and population pharmacokinetic modelling – an EINSTEIN JUNIOR phase I study. Thrombosis J. 2018. 10.1186/s12959-018-0185-1. 10.1186/s12959-018-0185-1 PMC6278136 30534008

[10] Yee DL, O'Brien SH, Young G (2013). Pharmacokinetics and pharmacodynamics of anticoagulants in paediatric patients. Clin Pharmacokinet.

[11] Corbella E, Miragliotta G, Masperi R, Villa S, Bini A, de Gaetano G (1979). Platelet aggregation and antithrombin III levels in diabetic children. Haemostasis.

[12] Fukui H, Taniguchi A, Sakamoto S, Kawahara S, Matsunaga T, Taira K (1989). Antithrombin III in children with various renal diseases. Pediatr Nephrol.

[13] Singh RR, Gupte-Singh KR, Wilson JP, Moffett BS (2016). Adherence to anticoagulant therapy in pediatric patients hospitalized with pulmonary embolism or deep vein thrombosis: a retrospective cohort study. Clin Appl Thromb Hemost.

[14] Halton JM, Lehr T, Cronin L, Lobmeyer MT, Haertter S, Belletrutti M (2016). Safety, tolerability and clinical pharmacology of dabigatran etexilate in adolescents. An open-label phase IIa study. Thromb Haemost.

[15] Halton JML, Albisetti M, Biss B, Bomgaars L, Brueckmann M, Gropper S (2017). Phase IIa study of dabigatran etexilate in children with venous thrombosis: pharmacokinetics, safety, and tolerability. J Thromb Haemost.

[16] Halton JML, Picard AC, Harper R, Huang F, Brueckmann M, Gropper S (2017). Pharmacokinetics, pharmacodynamics, safety and tolerability of dabigatran etexilate oral liquid formulation in infants with venous thromboembolism. Thromb Haemost.

[17] Young G, Yee DL, O'Brien SH, Khanna R, Barbour A, Nugent DJ (2011). FondaKIDS: a prospective pharmacokinetic and safety study of fondaparinux in children between 1 and 18 years of age. Pediatr Blood Cancer.

[18] Young G, Tarantino MD, Wohrley J, Weber LC, Belvedere M, Nugent DJ (2007). Pilot dose-finding and safety study of bivalirudin in infants <6 months of age with thrombosis. J Thromb Haemost.

[19] O'Brien SH, Yee DL, Lira J, Goldenberg NA, Young G (2015). UNBLOCK: an open-label, dose-finding, pharmacokinetic and safety study of bivalirudin in children with deep vein thrombosis. J Thromb Haemost.

[20] Forbes TJ, Hijazi ZM, Young G, Ringewald JM, Aquino PM, Vincent RN (2011). Pediatric catheterization laboratory anticoagulation with bivalirudin. Catheter Cardiovasc Interv.

[21] Young G, Boshkov LK, Sullivan JE, Raffini LJ, Cox DS, Boyle DA (2011). Argatroban therapy in pediatric patients requiring nonheparin anticoagulation: an open-label, safety, efficacy, and pharmacokinetic study. Pediatr Blood Cancer.

[22] Howie SRC (2011). Blood sample volumes in child health research: review of safe limits. Bull World Health Organ.

